# Performance analysis of multiple Indoor Positioning Systems in a healthcare environment

**DOI:** 10.1186/s12942-016-0034-z

**Published:** 2016-02-03

**Authors:** Tom Van Haute, Eli De Poorter, Pieter Crombez, Filip Lemic, Vlado Handziski, Niklas Wirström, Adam Wolisz, Thiemo Voigt, Ingrid Moerman

**Affiliations:** INTEC Department, IBCN, Ghent University, Gaston Crommenlaan 8, 9050 Ghent, Belgium; Televic NV, Leo Bekaertlaan 1, 8870 Izegem, Belgium; TUB, Straße des 17. Juni 135, 10623 Berlin, Germany; SICS, Isafjordsgatan 22, 164 40 Kista, Stockholm, Sweden

**Keywords:** Indoor localization, Healthcare, Hospital, RSSI, ToA, Fingerprinting

## Abstract

**Background:**

The combination of an aging population and nursing staff shortages implies the need for more advanced systems in the healthcare industry. Many key enablers for the optimization of healthcare systems require provisioning of location awareness for patients (e.g. with dementia), nurses, doctors, assets, etc. Therefore, many Indoor Positioning Systems (IPSs) will be indispensable in healthcare systems. However, although many IPSs have been proposed in literature, most of these have been evaluated in non-representative environments such as office buildings rather than in a hospital.

**Methods:**

To remedy this, the paper evaluates the performance of existing IPSs in an operational modern healthcare environment: the “Sint-Jozefs kliniek Izegem” hospital in Belgium. The evaluation (data-collecting and data-processing) is executed using a standardized methodology and evaluates the point accuracy, room accuracy and latency of multiple IPSs. To evaluate the solutions, the position of a stationary device was requested at 73 evaluation locations. By using the same evaluation locations for all IPSs the performance of all systems could objectively be compared.

**Results:**

Several trends can be identified such as the fact that Wi-Fi based fingerprinting solutions have the best accuracy result (point accuracy of 1.21 m and room accuracy of 98 %) however it requires calibration before use and needs 5.43 s to estimate the location. On the other hand, proximity based solutions (based on sensor nodes) are significantly cheaper to install, do not require calibration and still obtain acceptable room accuracy results.

**Conclusion:**

As a conclusion of this paper, Wi-Fi based solutions have the most potential for an indoor positioning service in case when accuracy is the most important metric. Applying the fingerprinting approach with an anchor installed in every two rooms is the preferred solution for a hospital environment.

## Background

In recent years, the complexity in nursing facilities has been increasing due to societal factors such as the increase of the care unit size, the increase of specialized care and the lack of nurse staffing, which requires a more efficient use of resources [1]. In addition to these inherent factors, a further increase in complexity is due to different technologies that is being introduced for the staff (e.g. medical equipment, pagers, alert redirecting and electronic medical records) as well as for the environment (e.g. building automation for energy control and comfort functions for the patients). In future years, these complexity trends will continue due to upcoming technologies (such as location aware services and computerized decision support systems) and an ageing society [2], which translates into an increasing need for care and a decrease of the available staff.

The introduction of location awareness in healthcare environments rises a wide range of new possibilities [3]. An Indoor Positioning System (IPS) allows hospitals to locate persons or assets inside the building. Interesting hospital scenarios could become reality: advanced nurse calling systems could locate the nearest nurse, making their work more efficient [4]. Patients with dementia will experience more freedom since they should not be locked away anymore. As a final example: finding assets inside a building can be a complicated task. In many cases time matters, finding an important asset faster can save lives. These are only a few examples how an IPS can improve the internal functionalities inside a hospital.

To avoid confusion, it is important to make a distinction between a “positioning“ and a “tracking” system. The latter uses history based information to estimate the location of the person or asset that needs to be tracked. This implies multiple negative consequences: (1) a start reference point is crucial when tracking is involved. If this is not calibrated carefully, tracking results will be useless. (2) The mobile node needs to communicate continuously to keep the location updated which will drain the battery much faster. (3) Finally, this causes conflicts in terms of privacy. When doctors or nurses are being tracked, their entire location history is available. Due to the concerns described previously, this paper focusses on Indoor Positioning Systems (IPSs). These systems determine the location of the mobile node only when it is requested. For locating a mobile node, an IPS uses multiple anchor nodes. This is comparable with the principle of Global Positioning System (GPS) for outdoor, which uses satellites and a GPS-receiver. Further details about IPSs are described in “Indoor Positioning Systems” section.

In scientific literature, a large number of IPSs has been proposed. Unfortunately, most of these have been evaluated in non-healthcare related environments using only point accuracy. As already mentioned, for many healthcare use cases, in addition to point accuracy other relevant metric need to be taken into account. Each of which can influence the choice of the optimal technology. Moreover, these metrics may vary depending on a particular environment. In other words, an evaluation in an operational hospital environment is imperative to be able to asses real-life localization performances.

The main contributions of this paper are as follows. (1) A performance evaluation of multiple wireless IPSs is performed in an operational hospital environment that was actively in use and as such has a representative deployment of Wi-Fi Access Points (APs) and typical hospital interference. (2) The impact of different design choices is quantified. The paper investigates the impact of the use of different localization algorithms, different wireless technologies and different anchor point locations. (3) The performance of the different set-ups is evaluated using multiple evaluation criteria, including point accuracy, room accuracy and latency. (4) The evaluation is focussed on stationary evaluation of localization solutions since the absence of history based location information is the most challenging. In this way, optimizations based on previous locations is excluded in the evaluation. (5) Finally, all data traces are made publicly available and can be used by third parties to evaluate additional IPSs.

The remainder of this paper is structured as follows. “Related work” section gives an overview of IPSs with their classification and which ones are suitable for healthcare environments. This section also discusses other research papers that compare and evaluate multiple solutions and technologies. “Methods” section describes the evaluation set-up, including the hospital environment, the used algorithms & hardware components and the evaluation methodology. Next, “Results and discussion” section discusses the performance evaluation for different set-ups and configurations. Finally, “Conclusion” section concludes the paper.

## Related work

### Indoor Positioning Systems

Due to satellite navigation systems, the rising trend of personal location-based services like guidance, tracking or navigation became possible [5]. However, the use of these satellite navigation systems (mainly GPS) is limited to outdoor environments, whereas many commercial applications are envisioned in indoor environments. To remedy this, IPSs are designed to meet the indoor requirements (also called “Indoor Localization Solutions”).

**Fig. 1:**
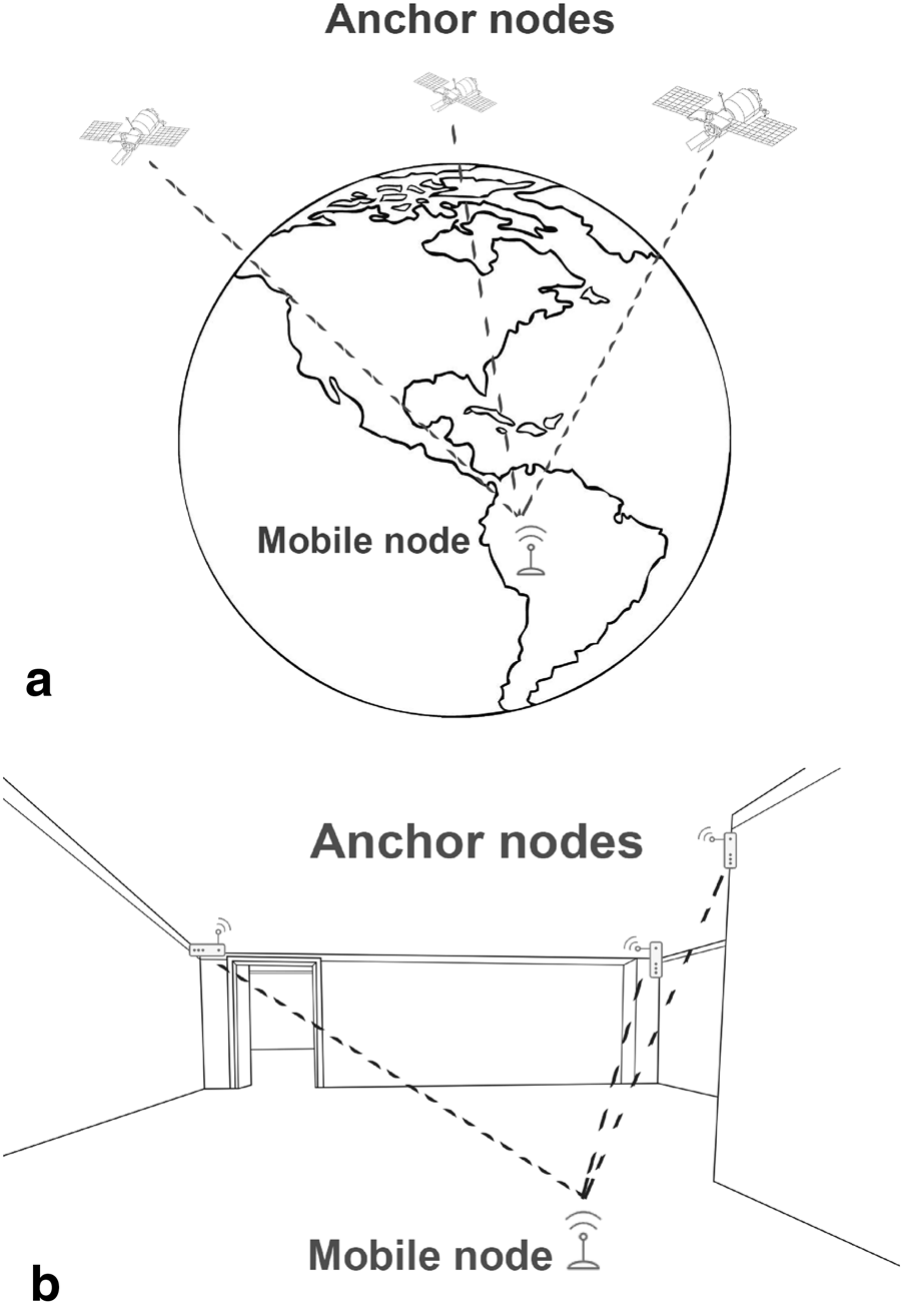
**a** The main principle of GPS illustrated, satellites act as anchor nodes broadcasting their signals to the mobile nodes. Mobile nodes receive those signals and estimate their position. In (**b**) a similar approach is illustrated indoor. *Anchor nodes* are placed inside a building which communicate with the mobile node

The main principle of GPS and IPS remains the same. This is illustrated in Fig. 1. Two types of nodes can be distinguished in the approach:

- *Mobile node(s)* The person or object that needs to be located, wears or is equipped with a mobile node. The solution calculates the location of this mobile device.
- *Anchor node(s)* In order to determine a location of the mobile node, multiple reference points are necessary. For outdoor navigation systems like GPS, satellites are used. Since their orbit is perfectly known, a GPS-receiver can calculate its own location based on the signals received from at least four satellites. For IPSs, the anchor nodes can be the existing Wi-Fi APs in the hospital or additionally installed nodes at tactical locations. Multiple technologies like Bluetooth Low Energy (BLE), Radio Frequency Identifier (RFID), ZigBee, etc. can be used.

**Fig. 2:**
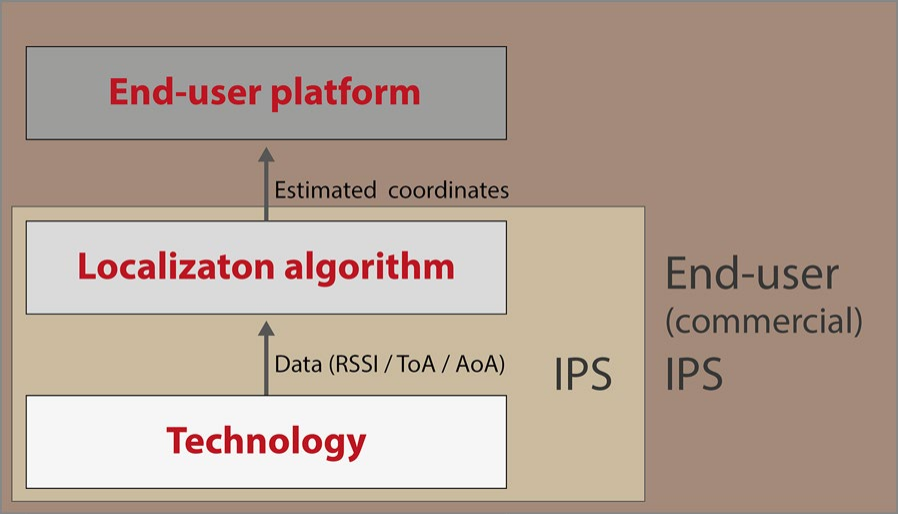
The three layers that define an end-user (commercial) IPS. The focus of this comparison are the two lower layers: the technology in combination with a certain localization algorithm. These two layers define an IPS whereby estimated coordinates of the mobile node are calculated

**Fig. 3:**
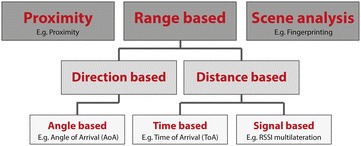
A classification of indoor localization solutions. Three categories are distinguished: proximity, range based and scene analysis. Range based can be split into direction or distance based. Direction based solutions uses the angle information of the antennas. Distance based solutions either use timing or signal based information

Typically, an IPS consists of an algorithm that processes wireless data from a specific technology. As such, an IPS can be seen as a combination of a localization algorithm running on top of a certain wireless hardware technology. Figure 2 presents the different layers. The main focus of this evaluation are the two lower layers: the technical performance. A localization algorithm can be classified in three categories as illustrated in Fig. 3.

- The principle of *proximity algorithms* [6] is locating a mobile node using the highest received signal strength of an anchor node. The mobile node (which is accompanied with the object or person that needs to be located) is in the proximity of this anchor node whereof the highest signal strength signal came from. Typically, near field communication (NFC) or RFID is applied for this approach. Although BLE is also capable to be used for proximity purposes. Proximity is easy to implement, does not require any complicated algorithms but the accuracy is low level, even room accuracy cannot be achieved. Since the accuracy is poor, this principle cannot meet the requirements from the hospital scenarios (described in the introduction).
- In contrast to proximity, *range based algorithms* use actual distances which are derived from the communication signals. A distinction between direction and distance based solutions can be made. Direction based means the direction of the propagation signal is the key element in determining the mobile node its position. Typically, an array of antennas or microphones is used to measure the angle between the signal and a reference. The spatial separation of antennas or microphones leads to differences in arrival times, amplitudes and phases. The most typical example is the “angle of arrival (AoA)”-approach [7]. It can achieve a high accuracy, however it includes a significant hardware cost. Therefore, it is not implemented often in commercial applications. Instead, algorithms based on the ranging distance are more popular. Two types can be differentiated: time or signal (property) based. Whilst time based algorithms (e.g. time of arrival (ToA) [8] and Time-difference of Arrival (TDoA)) determine distances based on the known signal propagation time, signal property based algorithms assume there is a proportional relationship between the Received Signal Strength Indicator (RSSI) and the distance. Generally, the main idea of range based algorithms remains the same: first, measured information (which may be derived from the angle, time or signal property information) is translated into a distance. Next, multiple distances are transformed into coordinates by applying Multilateration (MLAT).
- The final category in Fig. 3 is *“Scene Analysis”*. The most typical example is Fingerprinting [9], which has a completely different approach than the ones described previously. This process is twofold. The first step (also training or offline step) includes an extensive survey of the environment whereby a set of training fingerprints (wireless characteristics, RSSI values of all available anchor nodes) is collected and stored into the training database. Second, the “online phase” consists of the location estimation. The currently measured wireless characteristics are compared with the fingerprinting database entries. The entry that matches the best will be used as the current location of the mobile node. Though this method of working is very accurate, it also has drawbacks. Completing this survey for an entire hospital is labor-intensive: every m^2^ needs to be scanned and stored in a database. Even worse, environmental changes like moving a metal closet are impermissible and rescanning the environment is essential to keep the system accurate.

The goal of this paper is to identity which combinations of localization algorithms and wireless technologies are the most suited for hospital environments.

### Comparison of multiple indoor localization solutions

To determine which solution is best suited, multiple relevant metrics have to be taken into account.

- *Room accuracy*: The possibility to locate a stationary mobile node at room level. E.g. locating an important but rarely used medical device can be equipped with a mobile node. If the position of this device is requested, room (and thus also floor) information can be sufficient.
- *Latency*: The time between sending a location request and receiving the location information. To continue with the previous example: the time it takes starting when a staff member sends a location request to locate a mobile node (which can be carried with a patient or attached to a medical device) until the staff member receives the location information that was requested. Another example is the “emergency call”. When a patient pushed a mobile panic button, the latency of the localization solution can have an impact on the health status of the involved patient.
- *Installation time/cost*: Hospital environments are (almost) continuously operational, meaning the installation time must be reduced to the minimum. Can the existing network be reused or is new wiring necessary? Does the solution requires recalibrating or not? Answers to those questions are reflected in the installation time and cost metric.
- *Energy consumption*: This metric is particularly important for the mobile node. This value is equivalent with the life-time of the device. A minimum duration of the mobile node can be required by the hospital.

The papers described below discuss multiple evaluation criteria, however these IPSs are not evaluated in an operational hospital environment.

In [10], a comparison of multiple Radio Frequency (RF)-based indoor localization solutions in heterogeneous environments using multiple evaluation criteria is described. The authors conclude that the accuracy of the solutions depends strongly on the characteristics of the environment and that a fine grid of evaluation points is required for an objective comparison of solutions. Since the evaluation environments described in [10] consist of an office environment as well as an open industrial environment, this work motivates the need for extended evaluation testing in an operational healthcare environment.

Gu et al.  [11] compared indoor localization solutions with a special focus on the wireless personal networks. In their comprehensive survey, they evaluate numerous solutions which include both commercial products and research-oriented solutions. Their evaluation criteria consists of security and privacy, cost, performance, robustness, complexity, user preferences, commercial availability and limitations. Their conclusions are in the same line as [12], each solution uses a certain type of technology, has its design and works well under certain situations.

Boulos and Berry [3] compares multiple IPSs to implement in a healthcare environment. However, they discuss the higher levels of integrating an IPS in a hospital and their consequences: the impact and changes for the staff and patients, the Return On Investment (ROI) of an IPS, the possible risks when the system fails, etc. For those reasons, this paper is complementary to our work.

Finally, Vakili et al. [13] compared a commercial and custom-made tracking solution. Their comparison is comprehensive, using multiple evaluation criteria. Despite, these tracking solutions require manual actions from the users. Patients or nurses need to swipe a tag in front of a card-reader to indicate their entrance of the current room.

Taking into account the lessons learned from the related work, this paper will evaluate along multiple evaluation criteria for existing IPSs in an operational environment using a clearly defined methodology for objective evaluation.

### Indoor localization solutions for healthcare environments

In scientific literature, several indoor localization solutions are proposed for the next generation of advanced healthcare applications [14–19]. However, they are not evaluated using the metrics above.

In [14], an indoor localization algorithm is described based on RSSI measurements that is optimized especially for the healthcare environments. Their solution guarantees room level accuracy while avoiding heavy investments by reusing the existing nurse call network. This approach achieves a high scalability since the mobile nodes locate themselves.

Chen describes in [15] a dynamic indoor localization solution based on active RFID. His algorithm is based on a cost function associated with a shape constraint factor. The cost function consists of the similarity and disparity of signal strength between the tracking and reference tags, as well as geometrical correlation properties. Results show that the proposed algorithm provides considerable improvement in average estimation error as compared with existing methods.

Ropponen et al. [16] presents an improved version of the low-frequency indoor localization system that is located under the floor. They achieved a larger detection range and a more durable antenna laminate. The measured tag detection was 2 m. The tag location reliability of 96.3 % was verified with a practical test.

In [17], a wireless localization network for patient tracking is presented. The network can track the locations of the patient and monitor their physical status i.e. walking, running, etc. by measuring their inertial movement using a three axis accelerometer. The Fleck-3 platform [18] is used for the static nodes. In this paper, a comparison is made between their own packet delivery ratio and the CC2431 Location Engine that used RSSI. This paper lacks any performance results like accuracy or latency and is only focussed on the network layer of the application.

A final example is LAURA [19], it stands for LocAlization and Ubiquitous monitoRing of pAtients for healthcare support. This solution is also using the signal strength of the ZigBee standard combined with a particle filter. LAURA achieves, both with static and moving patients, an average localization error lower than 2 m in 80 % of the cases.

The mentioned papers above all describe a tracking solution designed and optimized for the healthcare sector. Some of them offer additional functionalities like patient monitoring. However, an objective evaluation approach is lacking. In many cases, no realistic hospital environment is used and multiple evaluation metrics like latency, installation cost, etc. are missing. This paper addresses these shortcomings.

## Methods

The next section describes in details the hospital environment, the used hardware, the localization algorithms and finally the measurement execution.

### Healthcare environment

For the measurement campaign, an actively used hospital environment (the Sint-Jozefskliniek hospital in Izegem, Belgium) was selected. The measurements were performed in the “surgical day hospital” ward, located in a new building on the first floor. In this particular ward, patients arrive in the morning to undergo surgery and leave at night. The end section of the corridor was available to perform the experiments, while the rest of ward was in “normal operation”, meaning patients and nurses were present and were walking around.

**Fig. 4:**
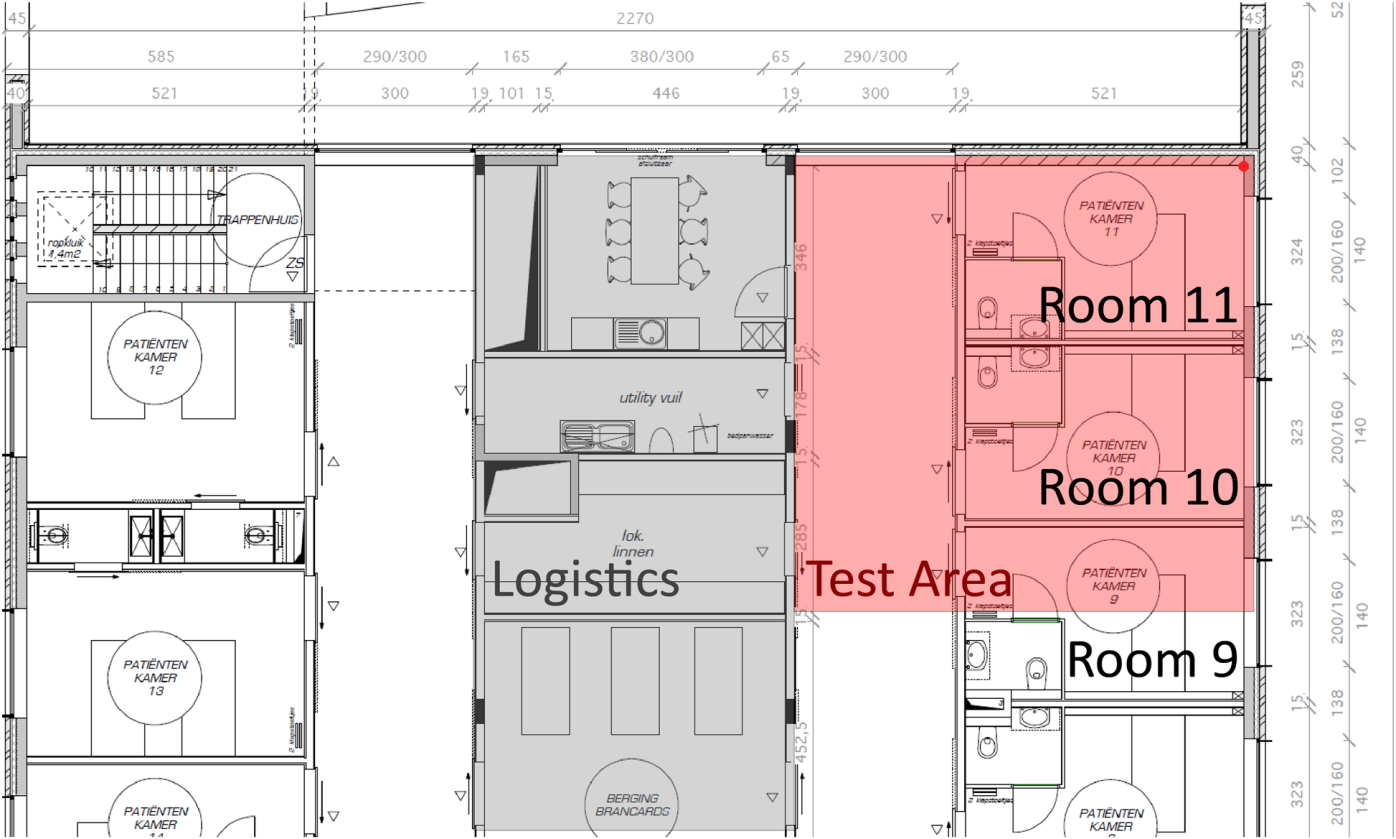
Floorplan of the hospital environment: rooms 9/10/11 and a hallway were used

The floor plan of the ward is depicted in Fig. 4. Rooms are located at both sides with “logistics” rooms in the middle. This means that there are two parallel corridors. Patient rooms 9, 10 and 11 were used for the evaluation. A dense evaluation grid of 1 m by 1 m was marked on the floor resulting in 73 evaluation locations where the position estimates were requested. Note that the grid was positioned in such a way that grid lines are 10 cm away from the wall. During the experiment, all doors were open.

### Installed hardware

**Fig. 5:**
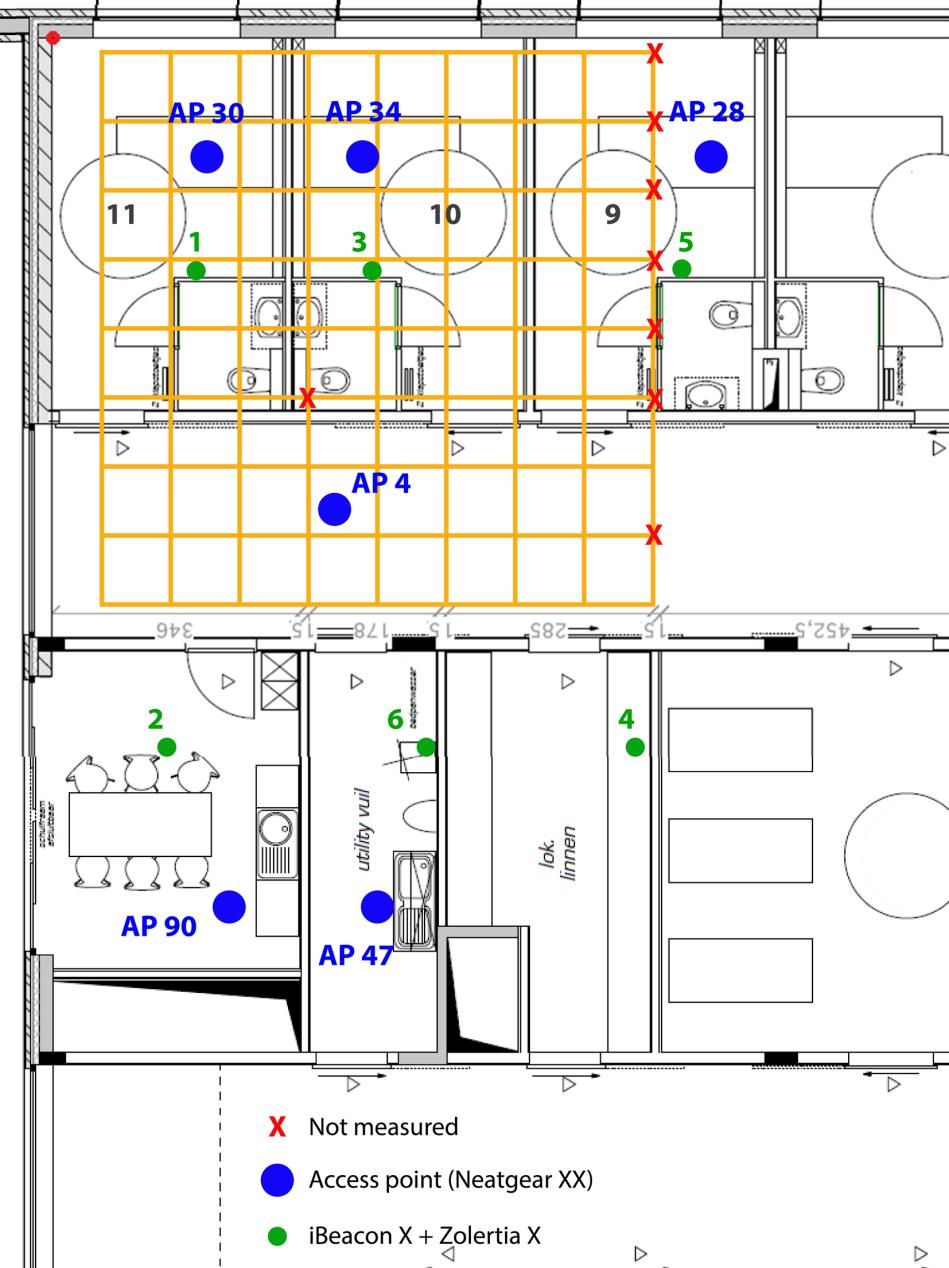
Floor plan of area in which the measurement campaign was performed. The evaluation points are located at the crossings of the *orange grid lines*. Deployed anchor points are indicated by *blue dots* (Wi-Fi access points) and *green dots* (ZigBee + BLE)

Anchor points from three different wireless technologies (Wi-Fi, ZigBee and BLE) were installed at the locations indicated on Fig. 5. The locations are selected as realistic as possible. Wi-Fi APs are placed on the ceiling above each bed, whilst the ZigBee and BLE nodes are placed on the wall nearby a light switch. Technical details of the devices can be found in Table 1.

**Table 1:** Technical information of the setup in the hospital environment

Technology	Technical details
Anchor points	
Wi-Fi	Netgear N750 Wireless Dual Band Gigabit Router
ZigBee	Zolertia Z1
BLE	BLE iBeacon (Estimote devices)
Mobile point	
Wi-Fi	External 300Mbps Mini Wireless N USB adapter: TL-WN823N (TP-Link)
ZigBee	External STM32W-RCFKIT (using channel 25 and TX output power 31, 0dBm)
BLE	External Belkin mini Bluetooth v4.0 Adapter

- *Wi-Fi* A set of six Wi-Fi APs were deployed. One in each room in our test area. These APs are marked with a blue dot in Fig. 5 (AP 30, 34, 28, 4, 90, 47).
- *ZigBee* A set of six ZigBee nodes (Zolertia Z1) were used during the measurement campaign. Their location is marked with a green dot in Fig. 5 (1, 2, 3, 4, 5, 6).
- *BLE* Each sensor node was accompanied with a BLE beacon.

### Localization algorithms

During the evaluation, three different localization approaches were evaluated: a scene analysis algorithm (fingerprinting), a time based algorithm (ToA) and finally a signal based algorithm (MLAT). A detailed description of the algorithms can be found in [20]. Figure 3 shows how the algorithms can be classified according to the classification from “Indoor Positioning Systems” section.

The fist solution is based on the fingerprinting principle. As mentioned in “Indoor Positioning Systems” section, this contains a twofold process whereby fingerprints are collected in a database during the learning phase. During the runtime phase, the current wireless statistics are compared and matched with the fingerprints in the database. In [21], the Wi-Fi network is used, but in theory any technology that contains RSSI values is possible. It is shown to be highly accurate, but it has drawbacks like installation and deployment time. This approach is also sensitive to changes in the environment. When this occurs, the training phase should be re-done.

A second approach is mainly based on the ToA-principle [22]. Time of arrival localization solutions estimate distances between devices based on the propagation time of an RF wave between sender and receiver. Using the measured time and the speed of light, a corresponding distance can be determined. It is expected that the propagation time is linear correlated with the distance. The number of clock ticks is measured how long it takes to receive an acknowledgement when an unicast message was transmitted to a certain node. This approach is combined with a particle filter and is called “Spray”. Since it mainly uses ToA information, this approach can only be evaluated using ZigBee data.

Finally an RSSI MLAT based algorithm [12]. This approach is only based on the linear relationship between the RSSI value of the signal and distance between sender and receiver. Firstly, distance estimations of at least three different anchor nodes are retrieved during the ranging phase. In the second step, MLAT is applied in order to estimate the mobile node its position. Like the fingerprinting method, it only requires RSSI values and thus each technology is suitable.

### Measurement execution

**Fig. 6:**
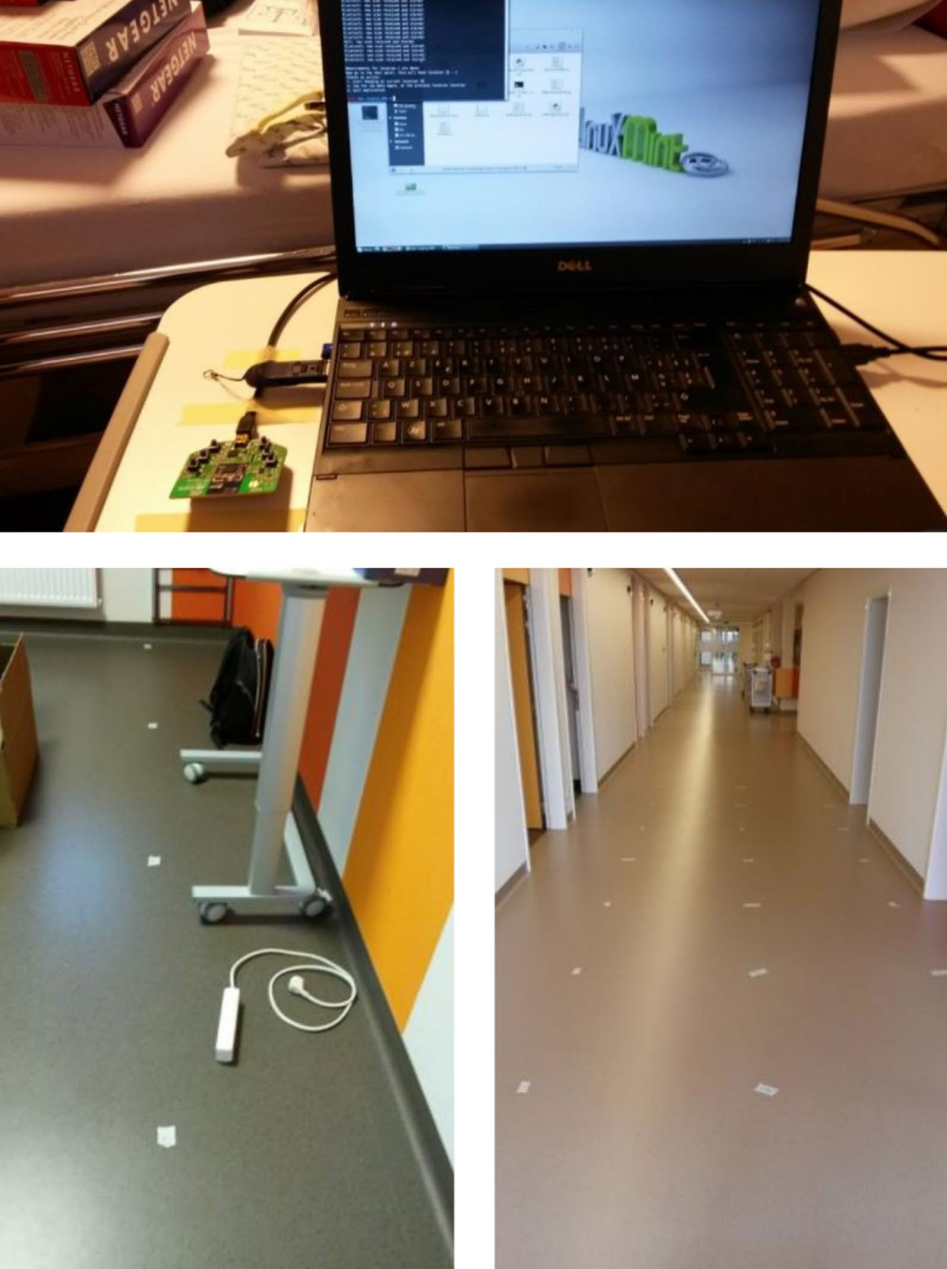
The mobile node: a Dell laptop with 3 dongles (Wi-Fi, BLE, sensor node STM32) at a trolley at 100 cm height. The ground truth of the evaluation points was indicated on the *white sticker* on the floor resulting in a gird of 1 m by 1 m. Picture of a room and the corridor

For the evaluation and comparison of different localization solutions the following approach is taken. (1) Packet transmitters (AP) of multiple technologies (Wi-Fi, ZigBee and BLE) are installed in an operational hospital environment. (2) A fine evaluation grid consisting of evaluation points with known locations is established and drawn on the floor of the hospital. (3) At each evaluation point packets from all APs are sampled. Since the measurement data is collected in an active hospital, with existing Wi-Fi access points as well as interference from other (medical) devices, realistic behaviour is obtained. (4) During the data capturing phase, information traces from multiple technologies are annotated and stored separately. The data was captured during 30 s with a laptop (moved around on a service cart) containing a dongle for each technology. An overview of the setup can be found in Fig.  6. The technical specifications of the used dongles can be found in Table 1. (5) In order to capture the influence of the number and locations of access points, filters are applied on the datasets whereby one or more access points are removed so the robustness of an algorithm can be determined. (6) Captured data is stored in the cloud and can be repeatedly used by a user to evaluate different algorithms. (7) Once the System Under Test (SUT) produced a set of estimates, a set of metrics are calculated as follows. For all IPSs, the position error, room error and latency were calculated in the 73 evaluation locations and afterwards averaged. These metrics were calculated using the evaluation criteria from the EVARILOS benchmarking handbook [23] which is aligned with the upcoming ISO/IEC JTC 1/SC 31 standard for evaluating RF-based IPSs.

Both the raw datasets and the metric results are publicly available on the EVARILOS benchmarking platform (EBP). The EBP was already extensively used on multiple events (EOC [24], IPSN [25], etc.) and it was shown to be useful for objectively capturing the performance and comparing multiple solutions using multiple evaluation metrics.

## Results and discussion

### Impact of the choice of the algorithms

**Fig. 7:**
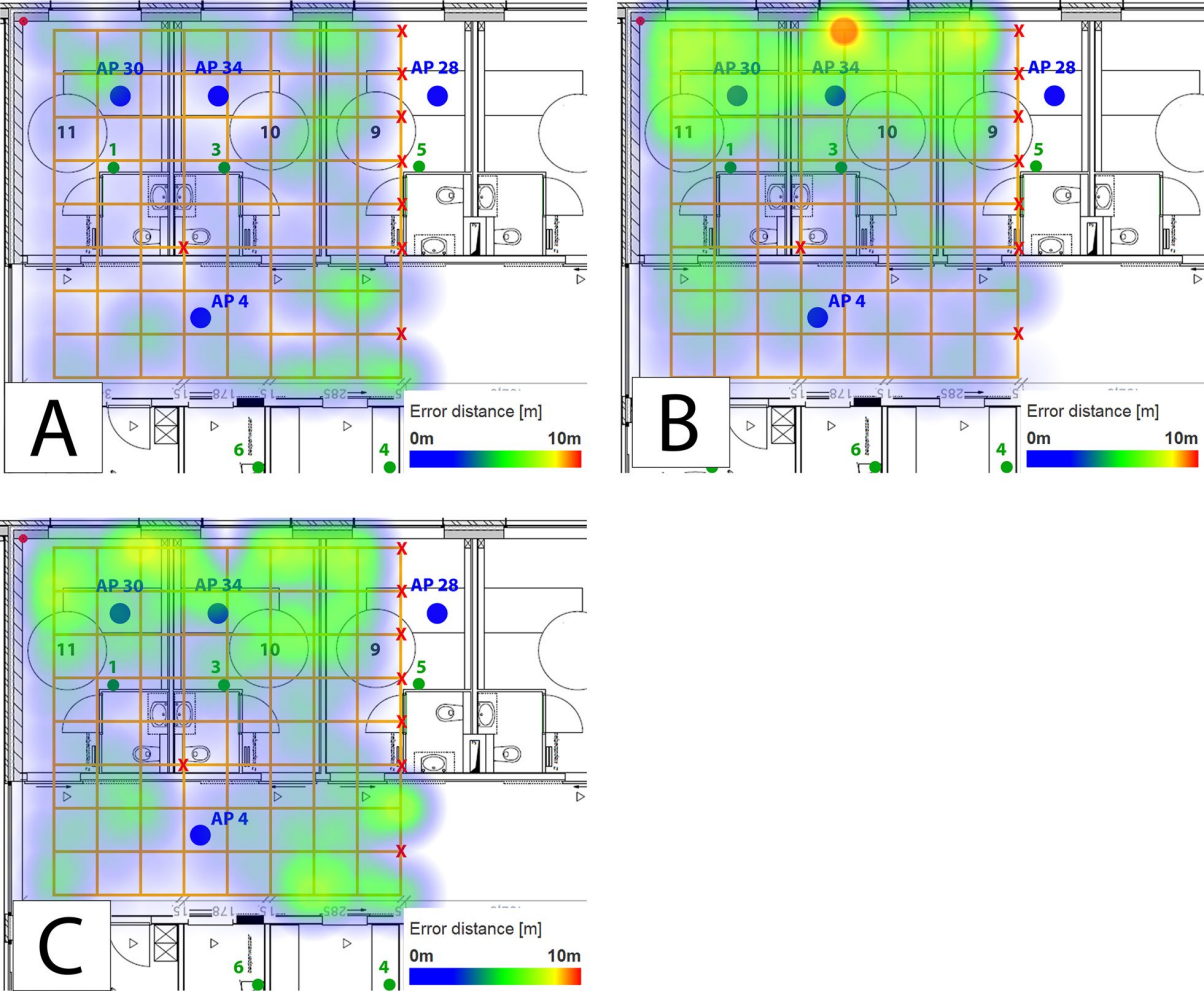
Heat maps representing the spatial distribution of localization errors of different localization algorithms using ZigBee data when all anchor nodes are used for location estimation. **A** Fingerprinting approach, **B** Spray approach, **C** MLAT approach

First, the performance results archived by different algorithms are compared to each other. For this evaluation, the data traces from all ZigBee node anchor points were given as input to all of the evaluated algorithms. ZigBee data is the only data source which may serve as input for all algorithms. The corresponding point accuracy is visualized in the form of heatmaps for each of the solutions in Fig. 7. Blue areas refer to good accuracy results (point accuracy of 2 m or less), whereas the accuracy worsens when the color changes to green, yellow and finally red. A red zone corresponds to a distance error around 10 m. A more detailed overview of the evaluation metrics using ZigBee can be found in Table 2.

**Table 2:** Comparison of the evaluation metrics using the ZigBee dataset

Algorithm	Point accuracy (m)	Room accuracy (%)	Latency (s)
Fingerprinting	1.99	88	1.65
RSSI MLAT	4.06	49	0.50
Spray (RSSI + ToA)	3.89	47	0.50

Based on Fig. 7, it is clear that fingerprinting approach achieves the most accurate results in general. The average error distance is 1.99 m. However, the latency is much higher than the one from other algorithms using the same data trace as input. In addition, fingerprinting solutions require a time-consuming calibration phase before they can be used, which might have to be repeated whenever the wireless environment changes significantly (for example due to the introduction of metal cupboards).

The spray solution is less accurate, achieving the average point accuracy around 3.89 m. In addition, in contrast to the previous solution, results show that the accuracy on one part of the environment is significantly higher than the accuracy in the other part. As a result, especially near the walls in the patient rooms, the corresponding room accuracy is significantly lower (Table 2).

Finally, the MLAT based approach is shown in Fig. 7C. The average accuracy is around 4.06 m. It is clear that the additional deployment costs for calibrating fingerprinting based solutions results in significantly better accuracy results.

### Impact of the choice of the technology

Wireless technologies like Wi-Fi, BLE or ZigBee have the common possibility to retrieve a measure of signal strength during the wireless communication: RSSI. Since RSSI values are used as input for two out of the three evaluated algorithms, this fact allows us to investigate the influence of the wireless technology on the accuracy of a localization algorithm. The stability or the variance of RSSI values often depends on the technology, since different technologies have different methods for calculating RSSI and are impacted differently by interference. As described in [26], Wi-Fi suffers from the coexistence of BLE and ZigBee (and vice versa), since they all operate in the 2.4 GHz ISM band.

The performance for each combination of technologies and algorithms is shown in Table 3. Since all algorithms use the same data trace as input, results can objectively be compared amongst different technologies and algorithms. Note that the spray algorithm requires time-of-arrival information, which is only available from the ZigBee nodes. As such, Spray is only evaluated using ZigBee data traces.

**Fig. 8:**
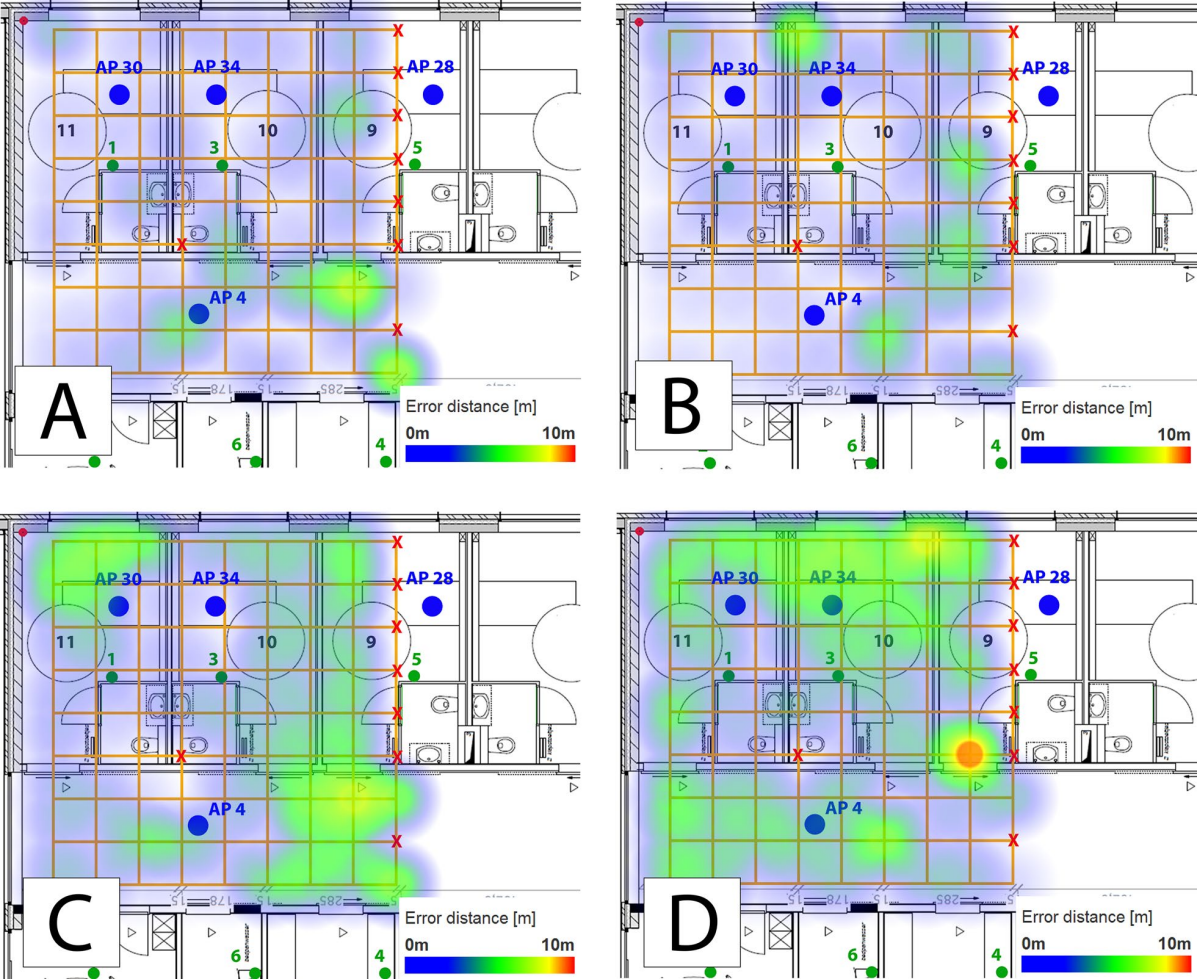
Comparison of point accuracy of different technologies and algorithms. **A** Fingerprinting, Wi-Fi; **B** Fingerprinting, BLE; **C** MLAT, Wi-Fi; **D** MLAT, BLE

The results of fingerprinting are shown in Fig. 8A and B. These are comparable with the results when ZigBee data was used (Fig. 7A). In general, this approach is very stable and achieve acceptable overall accuracy results. Further, no unexpected results are obtained for the MLAT approach (Fig. 8C, D). The error distances are comparable, except one outlier is detected when BLE data is used. These conclusions are reflected in the heat maps.

Table 3 shows that both solutions achieve the best accuracy results when Wi-Fi data is used. But at the same time Wi-Fi provides the worst latency results. One duty cycle for scanning for available networks takes 3 s. This cannot be interrupted.

As a conclusion, the differences between the technologies are minimal. Wi-Fi is slightly better and similar performance was achieved by ZigBee and BLE. A possible explanation is the difference in output power of these technologies. Wi-Fi’s output power is higher whilst the output power of ZigBee and BLE is quite similar.

### Impact of the choice of the anchor point selection

A final analysis will be discussed in this subsection: the influence of the available anchor points. Anchor points have a huge impact on installation time and robustness of the solution (in case an anchor point fails). To limit the number of heat maps, only the influence of access points with the MLAT algorithm is discussed.

In the previous sections, all available access points were used. The same datasets of the previous sections could be reused since additional filter techniques are applied. In this way, a perfect comparison is possible, since datasets contain the same interference and pedestrian pattern.

**Fig. 9:**
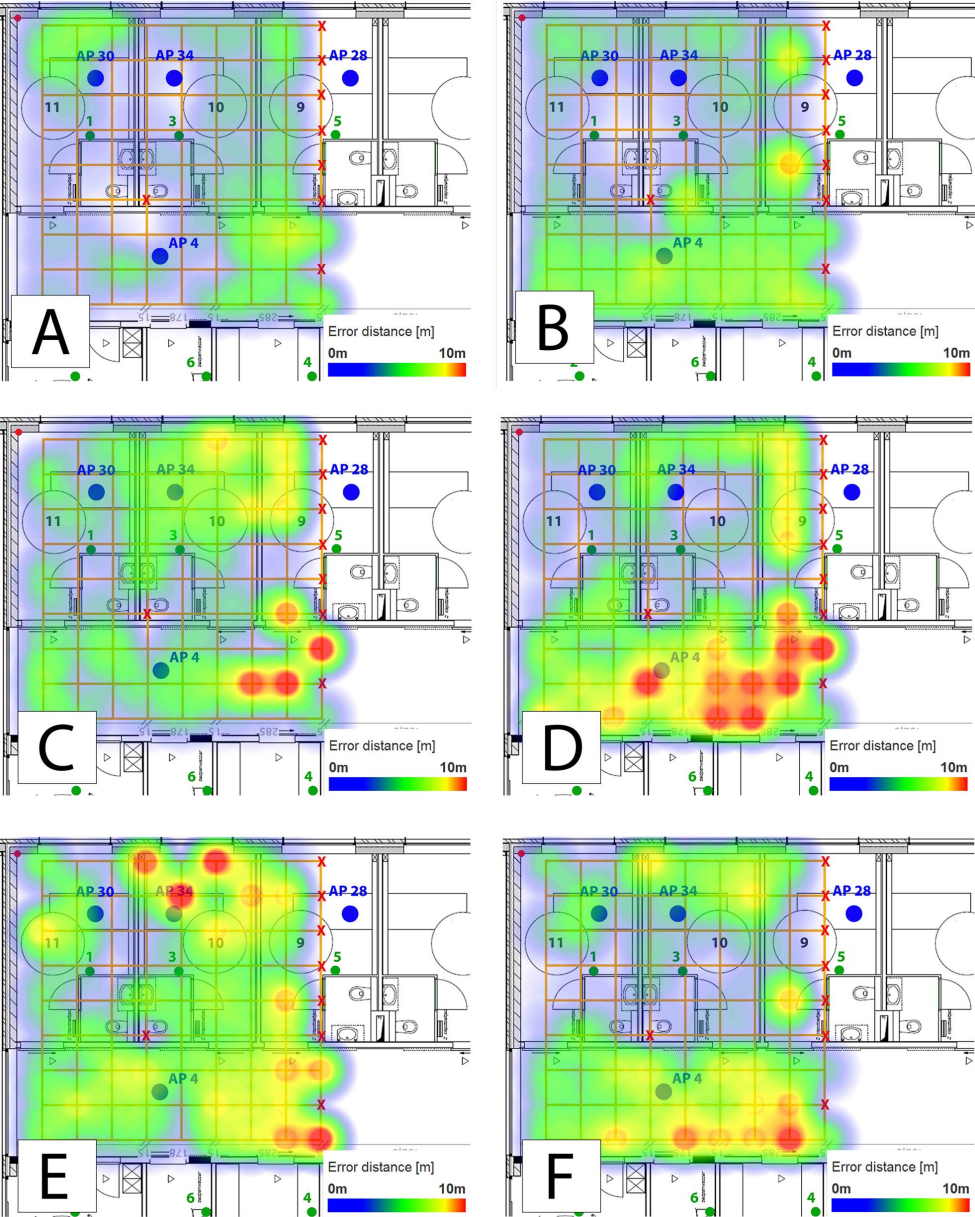
Heat maps representing the point accuracy of MLAT using all kind of data with different amount of anchor nodes. **A** Wi-Fi, without AP 4; **B** Wi-Fi, one side corridor; **C** BLE, only edges; **D** BLE, one side corridor; **E** ZigBee, only edges; **F** ZigBee, one side corridor

Figure 9 shows all the heat maps (of each technology) whereby a different set of anchor points is used. Initially all six anchor nodes were used, the location of these anchors can be found on the map in Fig. 5. For each technology, two different subsets are created. For Wi-Fi, the first one is without the centre AP located in the corridor and AP 4 (Fig. 9A). In this situation, the algorithm still achieves stable results. The changes are minimal compared with Fig. 8C). The error distances increase when only one side of the corridor is equipped with APs. In this case, AP 28, 30 and 34 are used. The solution performs weak mainly in the corridor and the third patient room. The point accuracy results remain more or less stable. On the other hand, the room accuracy drops drastically, from 56 to 31 % (Table 4).

The subset “Only edges” contains the anchors located in the corners of the evaluation environment (1, 2, 4 and 5). If BLE is the used technology, the algorithm has high error distances at the east side of the corridor (Fig. 9C). A big contrast when all anchor points are available (Fig. 8D). It is even worse when ZigBee data is used (Fig. 9E) The solution obtains a few error distances around 10 m at the east side of the corridor, but the worst results are retrieved at the top of the test area, in a patient room.

Figure 9D shows the spatial distribution of the localization error when using only the anchor points at the same side of the corridor (Anchor 1, 3 and 5). Higher errors can be observed in the corridor. In the patient rooms the algorithm works well as the evaluation points are in close proximity while in the corridor this is not the case. Having anchors on one line is clearly not a good option. For ZigBee, the same conclusion holds (Fig. 9F).

Anchor nodes have a strong influence on the MLAT algorithm. Explicable, since the approach is based on MLAT, results are worse if the anchors are positioned in one line instead of a triangle [27]. In comparison, fingerprinting remains more stable when certain anchor nodes are unavailable.

**Table 3:** Comparison of the evaluation metrics using the full dataset

Technology	Point accuracy (m)	Room accuracy (%)	Latency (s)
*Fingerprinting*			
ZigBee	1.99	88	1.65
Wi-Fi	1.21	96	5.43
BLE	2.13	79	3.06
*RSSI MLAT*			
ZigBee	4.06	49	0.50
Wi-Fi	3.65	47	3.00
BLE	3.85	61	2.50
*Spray RSSI + ToA*			
ZigBee	3.89	47	0.50

**Table 4:** Accuracy results with different subsets of anchor points

Filter	Point accuracy (m)			Room accuracy (%)
	Minimum	Average	Maximum	
*Wi-Fi*				
All anchors	0.52	2.68	5.95	46.58
Without AP4	0.07	2.69	6.77	56.16
One side corridor	0.48	3.47	8.57	31.51
*BLE*				
All anchors	0.94	3.12	9.09	61.64
Only edges	0.54	4.04	10.49	41.10
One side corridor	0.48	4.78	10.49	36.99
*ZigBee*				
All anchors	0.10	3.08	7.35	49.32
Only edges	0.48	5.09	10.89	21.92
One side corridor	0.48	4.24	9.94	38.36

### Discussion

When accuracy is the most deciding factor, fingerprinting lends itself to be the solution that should be implemented in combination with Wi-Fi access points. Moreover, fingerprinting proofs to be the most robust as well. When one of the APs fails, it will preserve its accuracy results. However when other parameters like installation time, “environment robustness” and latency have an influence on the decision, other approaches like Spray might become interesting.

## Conclusion

The need for more advanced systems is rising in the healthcare industry. Nurse calling or patient tracking systems need accurate and always up to date location information. Once this information is adopted in the previous mentioned systems, a whole of new services will become available. Therefore, in this paper, a thorough analysis of multiple facets of indoor localization approaches in an healthcare environment is executed.

Firstly, multiple algorithms are evaluated using the same amount and type of data. A fingerprinting, ToA and MLAT approach are compared using one single dataset, recorded in an operational hospital environment. Based on the accuracy results, fingerprinting achieves the best score (1.21 m). But on the other hand, fingerprinting has the highest latency and the worst installation and configuration time. A trade-off must be made depending on the primary requirements.

A second validation was the type of technology. Wi-Fi, BLE and ZigBee data was recorded during the measurement campaign in the hospital. The influence of the technology seems to be minimal on the accuracy metrics. However, latency impact is different: Wi-Fi has a duty cycle of 3 s. It takes at least 3 s before any RSSI data is available. BLE and ZigBee their update cycles are much shorter. In conclusion, when accuracy matters the most, Wi-Fi technology along with a fingerprinting algorithm yields the best result. The reason for that can be found in a higher bandwidth and transmission power of Wi-Fi in comparison to other technologies, which results in more stable RSSI measurements and higher coverage of Wi-Fi signals in the environment. When latency, power consumption or deployment matter the most, a “cheap” technology, such as BLE or ZigBee, are a decent alternative. When you can realize a dense deployment easily (at least one per room), the accuracy can be very good as well but more nodes are needed than in the Wi-Fi case.

Finally, an influence of the anchor nodes was evaluated. It is crucial to know how many anchor nodes are required for achieving accurate results and how a failure of one single anchor node can influence the stability of the entire system. Two different subsets are compared with the original situation. For the MLAT approach, the impact is of missing anchors is clearly visible at the heat maps. In case Wi-Fi is used, the area where the mobile node is localized, should be as much as possible within the anchor points. Optimally the resolution in X and Y directions is similar. This means that access points in every direction surround you. Additional anchors in the corridor are not required as they do not significantly improve the accuracy. Typically, one access point per every two rooms is a good compromise between accuracy and deployments. If ZigBee or BLE is used, a denser deployment is required than in the case of Wi-Fi. Of course these nodes are cheaper and consume less energy. A node per room is required. Nodes should be present in the rooms at both sides of the corridor. In that case, no additional nodes in the corridor are needed.

In general, Wi-Fi technology has most potential for cases where accuracy matters the most. The complexity of the algorithm is more important than the raw technology choice. ZigBee and BLE technologies show very similar results. A Wi-Fi fingerprinting solution with an anchor installed in every two rooms would be the preferred solution for a hospital environment.

## Abbreviations

AoA: angle of arrival
AP: access point
BLE: Bluetooth Low Energy
EBP: EVARILOS benchmarking platform
GPS: Global Positioning System
IPS: Indoor Positioning System
ISM: industrial, scientific and medical
MLAT: multilateration
NFC: near field communication
RF: Radio Frequency
RFID: Radio Frequency Identifier
ROI: Return On Investment
RSSI: Received Signal Strength Indicator
SUT: System Under Test
TDoA: Time-difference of Arrival
ToA: time of arrival

## References

[1] Buchan J, Aiken L (2008). Solving nursing shortages: a common priority. J Clin Nurs.

[2] Mann WC (2004). The aging population and its needs. IEEE Pervasive Comput.

[3] Boulos MNK, Berry G (2012). Real-time locating systems (RTLS) in healthcare: a condensed primer. Int J Health Geogr.

[4] Tucker AL, Spear SJ (2006). Operational failures and interruptions in hospital nursing. Health Serv Res.

[5] Drawil NM, Amar HM, Basir O (2013). Gps localization accuracy classification: a context-based approach. IEEE Trans Intell Transp Syst.

[6] Jiang X, Liang C-JM, Chen K, Zhang B, Hsu J, Liu J, Cao B, Zhao F. Design and evaluation of a wireless magnetic-based proximity detection platform for indoor applications. In: Proceedings of the 11th international conference on information processing in sensor networks, 2012. p. 221–232. ACM.

[7] Kułakowski P, Vales-Alonso J, Egea-Lóopez E, Ludwin W, García-Haro J (2010). Angle-of-arrival localization based on antenna arrays for wireless sensor networks. Comput Electr Eng.

[8] Chan Y-T, Tsui W-Y, So H-C, Ching P-c (2006). Time-of-arrival based localization under NLOS conditions. IEEE Trans Veh Technol.

[9] Meng W, Xiao W, Ni W, Xie L. Secure and robust wi-fi fingerprinting indoor localization. In: 2011 International conference on indoor positioning and indoor navigation (IPIN), 2011. p. 1–7. IEEE

[10] Van Haute T, De Poorter E, Moerman I, Lemic F, Handziski V, Adam W, Wirström N, Voigt T (2015). Comparability of RF-based indoor localization solutions in heterogeneous environments: an experimental study. Int J AdHoc Ubiquitous Comput.

[11] Gu Y, Lo A, Niemegeers I (2009). A survey of indoor positioning systems for wireless personal networks. IEEE Commun Surv Tutor.

[12] Van Haute T, Rossey J, Becue P, De Poorter E, Moerman I, Demeester P. A hybrid indoor localization solution using a generic architectural framework for sparse distributed wireless sensor networks. In: 2014 federated conference on computer science and information systems (FedCSIS), 2014. p. 1009–1015. IEEE

[13] Vakili S, Pandit R, Singman EL, Appelbaum J, Boland MV (2015). A comparison of commercial and custom-made electronic tracking systems to measure patient flow through an ambulatory clinic. Int J Health Geogr.

[14] Wyffels J, De Brabanter J, Crombez P, Verhoeve P, Nauwelaers B, De Strycker L (2014). Distributed, signal strength-based indoor localization algorithm for use in healthcare environments. IEEE J Biomed Health Inform.

[15] Chen W-H, Chang HH, Lin T-H, Chen P-C, Chen L, Hwan S, Yen D, Yuan HS, Chu WC. Dynamic indoor localization based on active rfid for healthcare applications: a shape constraint approach. In: 2nd international conference on biomedical engineering and informatics, 2009. BMEI’09, 2009. p. 1–5. IEEE.

[16] Ropponen A, Rimminen H, Sepponen R (2011). Robust system for indoor localisation and identification for the health care environment. Wireless Personal Commun.

[17] D’Souza M, Wark T, Ros M. Wireless localisation network for patient tracking. In: International conference on intelligent sensors, sensor networks and information processing, 2008. ISSNIP 2008. p. 79-84. IEEE.

[18] Sitka P, Corke P, Overs L, Valencia P, Wark T. Fleck-a platform for real-world outdoor sensor networks. In: 3rd international conference on intelligent sensors, sensor networks and information, 2007. ISSNIP 2007. p. 709–714. IEEE

[19] Redondi A, Tagliasacchi M, Cesana M, Borsani L, Tarrío P, Salice F. Lauralocalization and ubiquitous monitoring of patients for health care support. In: 2010 IEEE 21st international symposium on personal, indoor and mobile radio communications workshops (PIMRC Workshops), 2010. p. 218–222. IEEE

[20] Crombez P, Gesquiere J, Glorioso G, Villoute C, Yves W, Wirström N, Van Haute T, De Poorter E, Handziski V, Lemic F, Wolisz A. D4.2 report on the results of the real-life experiments in the validation scenarios. EVARILOS project.

[21] Lemic F. Benchmarking of quantile-based indoor fingerprinting algorithm. Telecommunication Networks Group, Technische Universität Berlin, Tech. Rep. TKN-14-001 (2014).

[22] Wirstrom N, Misra P, Voigt T. Spray: a multi-modal localization system for stationary sensor network deployment. In: 2014 11th annual conference on wireless on-demand network systems and services (WONS), 2014. p. 25–32. IEEE.

[23] Van Haute T, De Poorter E, Lemic F, Handziski V, Voigt T, Wolisz A, Moerman I (2015). Platform for benchmarking of RF-based indoor localization solutions. IEEE Commun Mag.

[24] Lemic F, Handziski V, Wolisz A, Constambeys T, Laoudias C, Adler S, Schmitt S, Yang Y. Experimental evaluation of RF-based indoor localization algorithms under RF interference. In: Proceedings of ICL-GNSS’15.

[25] Lymberopoulos D, Liu J, Yang X, Choudhury RR, Handziski V, Sen S. A realistic evaluation and comparison of indoor location technologies: experiences and lessons learned. In: Proceedings of the 14th international conference on information processing in sensor networks, 2015. p. 178–189. IPSN ’15.

[26] Pei L, Liu J, Guinness R, Chen Y, Kroger T, Chen R, Chen L. The evaluation of wifi positioning in a bluetooth and wifi coexistence environment. In: Ubiquitous positioning, indoor navigation, and location based service (UPINLBS), 2012. p. 1–6 (2012). IEEE.

[27] AbdelSalam HS, Olariu S. Towards enhanced RSSI-based distance measurements and localization in WSNs. In: INFOCOM Workshops 2009, IEEE, 2009. p. 1–2. IEEE.

